# Relationship Between Proactive Personality and Entrepreneurial Intentions in College Students: Mediation Effects of Social Capital and Human Capital

**DOI:** 10.3389/fpsyg.2022.861447

**Published:** 2022-06-16

**Authors:** Ya-Fei Luo, Jianhao Huang, Sunyu Gao

**Affiliations:** ^1^Hengshui University, Hengshui, China; ^2^Dhurakij Pundit University, Bangkok, Thailand

**Keywords:** proactive personality, entrepreneurial intention, social capital, human capital, chain mediation effect

## Abstract

The research aims to explore the influence mechanism of proactive personality on the entrepreneurial intentions of college students. Adopting proactive personality scale, social capital scale, human capital scale, and entrepreneurial intention scale, this research tested valid samples of 300 Chinese college students. The results revealed that proactive personality exerted a significant and positive impact on the entrepreneurial intentions. Social capital played a partial mediating role between the proactive personality and the entrepreneurial intentions. Human capital also played a partial mediating role between proactive personality and entrepreneurial intentions. Social capital and human capital exerted a chain mediation effect between proactive personality and entrepreneurial intentions. The result of this research offers valuable insights to the study of the influence of college students’ proactive personality on entrepreneurial intentions and provides entrepreneurship education management in colleges with specific practical suggestions so as to improve entrepreneurial intentions among college students.

## Introduction

Entrepreneurship can bring technological innovation and organizational breakthroughs, alleviate employment pressure, and promote economic development ultimately (Nguyen, 2021). Colleges are important talent pools of future entrepreneurs, and it was necessary to attach importance to entrepreneurial management to alleviate the employment pressure of college students (Lipkind and Kitrar, 2021). According to studies, the entrepreneurial behavior of college students was fundamentally influenced by their entrepreneurial intentions (Bird, 1988; Krueger, 2007). However, some researchers showed that the entrepreneurial intentions of college students were further improved (Peng et al., 2019). Therefore, exploring the key factor of entrepreneurial intentions of college students is currently an important research issue.

Studies revealed personality traits as important predictors of entrepreneurial intention among various influencing factors related to entrepreneurial intention (Bateman and Crant, 1993; Wu et al., 2019). It was reported that compared to individuals with low levels of proactive personality, the individuals with high levels of proactive personality exhibited greater compatibility with entrepreneurial activities (Holland, 1987). According to Zareieshamsabadi et al. (2010), proactive personality significantly affected employees’ entrepreneurial intentions. Nowadays, there were similar research results in the educational field, showing that the proactive personality of college students had a significant and positive effect on their entrepreneurial intentions (Zeb et al., 2019). Basar (2017) observed that college students with a proactive personality were better at discovering and capturing opportunities, which ultimately assisted them in developing entrepreneurial intentions and, therefore, gaining a competitive advantage over other students. However, studies confirmed that proactive personality could improve entrepreneurial intention, educational organizations possessing the nature of non-profit institutions, showing different characteristics with business organizations. Consequently, taking college students as a research object, this research explores the relationship between proactive personality and entrepreneurial intentions in the hope of obtaining more empirical research evidence.

Based on the social capital theory (Burt, 1995), entrepreneurs obtained resource support by using social capital and thus improved their entrepreneurial intentions (Hsiao et al., 2016). Meanwhile, based on the human capital theory (Schultz, 1961), the human capital accessible to entrepreneurs was the main factor in deciding whether to start a business (Ho et al., 2010). According to the current empirical study, this research noticed that proactive personality had a significant and positive impact on social capital (Nasaj, 2021). Social capital had a significant and positive impact on entrepreneurial intention (Mahfud et al., 2020). Proactive personality had a significant and positive impact on human capital (Seibert et al., 1999). Human capital had a significant and positive impact on entrepreneurial intention (Moradi et al., 2014). Moreover, social capital and human capital were not isolated (Gradstein and Justman, 2000). Social capital had a significant and positive impact on human capital (Wang, 2021).

In summary, existing researches show that proactive personality has a significant and positive impact on entrepreneurial intention, and social capital and human capital have mediation effect on proactive personality and entrepreneurial intention. However, the internal mechanism of social capital and human capital between proactive personality and entrepreneurial intentions of college students is still not clear in educational literature. Especially, colleges are important places to accumulate social capital as well as knowledge and skills. The social and training service colleges may improve college students’ social capital and human capital. College students’ entrepreneurial intentions may be enhanced due to the improvement of their proactive personality. This is an important task for college innovation and entrepreneurship education management. In this view, this research assumes that social capital and human capital may be important mediating variables between proactive personality and college students’ entrepreneurial intentions.

Therefore, this research explores the influence of college students’ proactive personality on entrepreneurial intentions and the mediation effect of social capital and human capital between proactive personality and entrepreneurial intentions, combining social capital theory and human capital theory. This research will help us further understand the important factors influencing the entrepreneurial intentions of college students to improve our knowledge of the potential influencing mechanisms underlying the process and provide colleges with a new direction of promoting college students’ entrepreneurial intentions more effectively.

## Literature Review

### Proactive Personality and Entrepreneurial Intention

“Proactivity refers to active attempts made by the individual to effect changes in his or her environment” (Zampetakis, 2008). Proactive personality refers to a stable and active personality that challenges the status quo by improving the existing environment or creating an entirely new environment rather than passively adapting to the current conditions (Crant and Bateman, 2000). In other words, individuals with a strong proactive personality could identify and exploit opportunities by demonstrating proactive, active, and persistent behaviors until the intended or certain meaningful changes were realized (Bateman and Crant, 1993). Prior research has found that people who are more proactive have higher entrepreneurial intentions (Crant, 1995). According to Thompson (2009), previous entrepreneurial intention refers to the notion that an individual intended to begin a new business and consciously planned to execute it at a certain point in the future. Entrepreneurial intention reflected a subjective intention of an individual during the entrepreneurial process and, therefore, served as a suitable predictor of entrepreneurial behaviors (Bird, 1988). Entrepreneurial intention, an individual’s intent to engage in entrepreneurial behavior, is central to understanding entrepreneurial activity in society (Krueger and Brazeal, 1994). Entrepreneurial intention also emphasized personal innovation, initiatives, and a degree of risk-bearing in an individual (Covin and Slevin, 1989). The previous empirical studies showed that personality traits had a significant impact on the entrepreneurial intention of college students (Cai et al., 2021; Jiatong et al., 2021; Murad et al., 2021). As a positive personality trait, individuals with proactive personality were more prone to starting a business. Existing studies demonstrated that employees’ proactive personality had a significant and positive impact on their entrepreneurial intentions (Zareieshamsabadi et al., 2010; Paul and Shrivatava, 2016). Researchers found out that, in the educational research field, college students’ proactive personality significantly impacted entrepreneurial intentions (Zeb et al., 2019; Hossain and Asheq, 2020). Some other researchers took college students in Istanbul as research objects, and found that proactive personality exerted positively predict college their entrepreneurial intentions (Basar, 2017). Some researchers took Chinese college students as the research object and the results showed that proactive personality had improved their entrepreneurial intention and further effectively transformed it into entrepreneurial behavior (Li et al., 2020a). It showed that the higher the level of proactive personality was, the greater the entrepreneurial intention would be. Therefore, this research proposes that H1: college students’ proactive personality has a significant and positive impact on entrepreneurial intentions.

### The Mediating Role of Social Capital Between Proactive Personality and Entrepreneurial Intention

Social capital was the resource comprising interpersonal relationships established by individuals through social relationships and networks (Coleman, 1988). Yang et al. (2011) proposed that the quality of undertaking initiatives was one of the important personality traits that promoted the accumulation of social capital. It was because when individuals with proactive personality faced difficulties and challenges, they would ask for help from others in their social network to expand social capital (Sharifian et al., 2022). Previous research also showed that the high level of proactive personality was beneficial to stimulating individuals’ social networking awareness to significantly and positively predict individuals’ social capital (Tang, 2016). In educational literature, the research of Le and Lin (2021) showed that college graduates’ proactive personality could significantly and positively influence the scale and behavior of their social network, and social network was an important content of social capital (Coleman, 1988). Therefore, college students’ proactive personality may significantly and positively impact social capital.

According to the social capital theory, the more interpersonal relationships individuals had to facilitate their actions; the more were their interpersonal relationships. They could utilize these relationships to gain actual or potential resources, and consequently, the greater was the social efficiency (Nahapiet and Ghoshal, 1998). In other words, individuals obtain entrepreneurial resources through social interpersonal relationships to increase the chances of starting a business (Weiss et al., 2019). Subramaniam and Youndt (2005) stated that social capital was one of the most important determinants of entrepreneurship. In comparison to individuals with low social capital, individuals with high social capital exhibited a stronger positive effect on their entrepreneurial intentions (Lee et al., 2014). Certain researchers proposed that social capital was an important condition for developing social entrepreneurial intention (Lan and Luc, 2020). In educational literature, Mahfud et al. (2020) established a structural model for the relationship of entrepreneurial intentions and concluded that social capital exerted a significant and positive effect on entrepreneurial intentions in vocational school students. A large number of studies confirmed that social capital is the significant predictive factor of students’ entrepreneurial intentions (Vuković et al., 2017; Pérez-Macías et al., 2021). Therefore, it may be inferred that the higher the social capital of college students is, the greater their entrepreneurial intentions will be.

This research concludes that a proactive personality may increase the social capital gained by college students, which will, in turn, significantly enhance their entrepreneurial intentions. When investigating the mediating relationship between proactive personality and work performance, Thompson (2005) expressed that employee with proactive personality obtained work performance benefits by developing social capital. Other studies demonstrated that social capital plays a mediating role in the relationship between proactive personality and turnover intention (Yang et al., 2011). It showed that social capital played a crucial mediating role in previous empirical research. Therefore, this research proposes that H2: social capital has a mediation effect in the influence of college students’ proactive personality on their entrepreneurial intentions.

### The Mediating Role of Human Capital Between Proactive Personality and Entrepreneurial Intention

Human capital was the total of various skills, knowledge, and quality of health, and experience that may collectively develop the economic value of the human body (Schultz, 1961; Subramaniam and Youndt, 2005). Basar (2017) proposed that individuals with a proactive personality actively accumulated human capital as they were more willing to learn and enhance their abilities and knowledge when encountering challenges to resolve problems (Gao et al., 2020). According to studies, the higher the level of proactive personality was, the higher the human capital would be, and vice versa (Demirtas et al., 2017). Seibert et al. (1999) proved that the parameter of proactive personality could positively predict human capital. In educational literature, the researcher discovered that proactive personality had a significant and positive influence on college students’ academic record (Ng et al., 2019), an important measure of students’ human capital (Rosen, 1989). In this view, students’ proactive personality may have a significant and positive impact on human capital, meaning that the higher level the college students’ proactive personality is, the more abundant their human capital would be.

Additionally, the human capital theory stated that the ability to study further assisted individuals in acquiring outstanding recognition capabilities, which enabled them to exhibit higher productivity and efficiency in a range of activities (Becker et al., 1980). In this view, human capital may facilitate the productivity of entrepreneurial activities. The higher the education and experience of individuals were, the higher their entrepreneurial intentions would be (Kim et al., 2006). It had been further demonstrated that knowledge management practices had a significant and positive impact on entrepreneurial performance (Li et al., 2020b). Stuetzer et al. (2013) also proposed that human capital positively impacted entrepreneurship. Combining human capital theory, Miao et al. (2015) discovered that human capital had a significant and positive impact on the entrepreneurial intention of overseas returnees. In the educational field, human capital, including education, experience, and training, was the leading factor in forming college students’ entrepreneurial intentions (Zhang et al., 2020). Human capital had a significant and positive impact on Chinese college students’ entrepreneurial intentions (Moradi et al., 2014). It indicated that when college students had a high level of human capital, they may have greater entrepreneurial intentions.

The present study, therefore, concludes that the proactive personality of college students might increase human capital, which will, in turn, significantly affect the entrepreneurial intentions among these students. Human capital was reported to play a crucial mediating role between international expatriation and entrepreneurial intention (Schlepphorst et al., 2020). Khoshmaram et al. (2020) reported that environmental support exerted a significant impact on entrepreneurial behavior *via* human capital. These evidence-based studies confirmed the mediating role of human capital. Therefore, this research proposes that H3: human capital has a mediation effect in the influence of college students’ proactive personality on their entrepreneurial intentions.

### The Chain Mediating Role of Social Capital and Human Capital Between Proactive Personality and Entrepreneurial Intention

Human capital theory and social capital theory had independent development and formation paths, but there were two different views in entrepreneurship research (Bozeman and Mangematin, 2004). Researchers noticed that the two might have a positive relationship (Serageldin and Dasgupta, 2001). Coleman (1988) pointed out that social capital is generated in people’s relationship with one another, and social capital within families (such as the relationship between parents and offspring), social capital outside families (such as community and neighborhood factors), and social capital as public goods (such as geographical environment and trust among people) all exerted an important impact on the formation of human capital. From this point of view, the social capital of entrepreneurs was profoundly influencing the formation and development of their future human capital. From another point of view, Mosey and Wright (2007) proposed that the difference in entrepreneurs’ social capital depended on whether they had previous working experience and entrepreneurial experience, which was actually their human capital. Those with related working experience generally could obtain more social capital. Therefore, it was beneficial for entrepreneurs with more abundant human capital to form and develop social capital.

However, “Guan xi” was a widespread phenomenon in China, which was even considered a part of Chinese culture so that could not be eliminated fundamentally (Hwang, 1987). “Chinese-style relationship” represented the “Guan xi” phenomenon unique to China (Chen and Chen, 2004). By analyzing the social network relationship of Chinese when they look for jobs, Bian (1997) discovered that “Chinese-style relationship” exerted a more important effect during employment. The social network relationship at workplaces better helped Chinese employees to achieve higher career achievements (Farh et al., 1998). One Chinese empirical study showed that social entrepreneurs’ successes relied greatly on their social network relationship (Wang et al., 2007). It indicated that “Chinese-style relationship” was undoubtedly important in Chinese society. Therefore, based on China’s cultural background, this research believed that the social capital of Chinese college students had a more profound influence on the formation and development of their human capital compared to college students in western countries.

Davidsson and Honig (2003) verified that social capital and human capital could both promote the establishment of new startups. Other researchers also discovered that social capital and human capital both had a significant and positive influence on college students’ entrepreneurial intentions (Jemari et al., 2017). One recent Chinese empirical research showed that the more college students’ social capital was, the higher their human capital would be. College students’ social capital even had a significant and increasing influence on their entrepreneurial intentions through the mediation effect of human capital (Wang, 2021). Another empirical research exploring employees’ personality traits and entrepreneurship influence mechanism demonstrated that social capital and human capital had important mediation effect on internal control personality and entrepreneurship (Hsiao et al., 2016). It can be thus inferred that college students’ social capital and human capital may play an important mediating role in proactive personality and entrepreneurial intentions.

To be more specific, Chinese college students’ proactive personality may improve their social capital and human capital, while social capital may also promote human capital to enhance their entrepreneurial intentions. Therefore, this research proposes that H4: social capital and human capital have a chain mediating role in influencing college students’ proactive personality on their entrepreneurial intentions.

## Materials and Methods

### Research Framework

**Figure 1:**
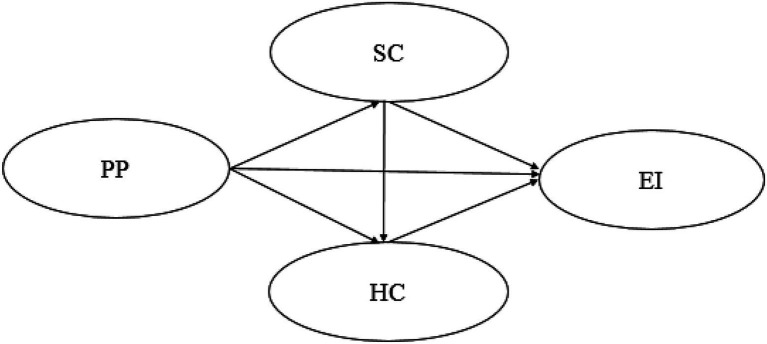
Research framework. PP, Proactive Personality; EI, Entrepreneurial Intention; SC, Social Capital; and HC, Human Capital.

This research took proactive personality as the independent variable and entrepreneurial intention as the dependent variable to explore the relationship between them, and the chain mediation effect of social capital and human capital in this relationship (Figure 1).

### Data Collection and Procedures

Using the convenient sampling method, the study conducted a questionnaire survey on students at a University in Hebei Province, China, on an online questionnaire platform. The selected university is a truly representative sample because the university is one of the demonstration bases of innovation and entrepreneurship education reform in China. In November 2021, teachers at the administration office sent the questionnaires in electronic forms to the students at our request. It took a university student approximately 30 min to fill in the questionnaire. The study distributed 310 questionnaires and received 300 valid ones after eliminating invalid ones, with a 97% response rate. Among the students who returned the valid questionnaires, there were 64 boys (21.3%) and 236 girls (78.7%), including 64 freshmen (21.3%), 164 sophomores (54.7%), 43 juniors (14.3%), and 29 seniors (9.7%). Among them, 85 majors in human resources management (28.3%); 55 majors in international economy and trade (18.3%); 68 majors in information system of department of information management (22.7%), and 92 from other majors (30.7%). The 300 students had participated in entrepreneurship courses or entrepreneurship training activities offered by colleges (100%). According to the formula provided by Israel (1992) for calculating the sample size, that is, sample size = *z*^2^ × *p*(1−*p*)/*e*^2^/1 + (*z*^2^ × *p*(1−*p*)/*e*^2^*N*)，*z* = 1.65, *p* = 0.5, *N* = 15,325, and *e*^2^ = 0.0025, the formal sample size should be no less than 268. The size of this study met the sampling criteria. The study was conducted in accordance with the Declaration of Helsinki and all subjects were willing to cooperate and had signed informed consent forms. The study gave due consideration to their privacy and wishes and informed them that they could refuse to participate or withdraw from the study at any time (Goodyear et al., 2007).

The analysis of this research consists of two steps: pilot test and formal test. In the pilot test stage, 114 valid questionnaires were collected and SPSS 21.0 was used to conduct item analysis, Exploratory Factor Analysis (EFA) and reliability analysis on these data to test the reliability and validity of the scale. In the formal test stage, 300 valid questionnaires were collected and SPSS 21.0 was used for descriptive statistics and correlation analysis of these data. In addition, AMOS 24.0 was used for measurement model and structural model tests.

### Measures

The questionnaire of this research includes four scales: proactive personality scale, social capital scale, human capital scale, and entrepreneurial intention scale, and pilot test data are adopted to make item analysis. The criteria for item analysis are that the *t* value of each item is greater than the reference value 3 (McIver and Carmines, 1981).

#### Proactive Personality Scale

The proactive personality scale revised by Shang and Gan (2009) was used. It was a seven-point scoring scale, with the scores ranging from 1 (strongly disagree) to 7 (strongly agree). A higher score indicated a level of proactive personality. The original scale comprised 11 items with no reverse item with a single dimension. The items were further screened through item analysis, and accordingly, one item was deleted, which was as follows—“If I see others in difficulty, I will try my best to help.” A total of 10 items remained finally.

#### Social Capital Scale

The human capital scale compiled by Snell and Dean (1992) was adopted. It was a seven-point scoring scale, with the scores ranging from 1 (strongly disagree) to 7 (strongly agree). A higher score indicated higher social capital. With a single dimension, the original scale comprised five items with no reverse item. The items were further screened through item analysis, based on which one item was deleted—“I can apply the knowledge from one field to the problems and opportunities in another field.” Finally, a total of four items remained.

#### Human Capital Scale

The human capital scale compiled by Snell and Dean (1992) was adopted. It was a seven-point scoring system, with the scores ranging from 1 (strongly disagree) to 7 (strongly agree). A higher score indicated higher human capital. With a single dimension, the scale comprised five items with no reverse item. In the further screening of items through item analysis, no items were required to be deleted.

#### Entrepreneurial Intention Scale

The entrepreneurial intention scale compiled by Liñán and Chen (2009) was adopted. It was a seven-point scoring scale, with the scores ranging from 1 (strongly disagree) to 7 (strongly agree). A higher score indicated greater entrepreneurial intention. With a single dimension, the scale comprised six items with no reverse item. When the items were further screened through item analysis, no items had to be deleted.

### Exploratory Factor Analysis

As visible in Table 1, this research used pilot test data to conduct EFA. According to the result, Kaiser-Meyer-Olkin (KMO) Test = 0.838, the significance of Bartlett’s Test of Sphericity value was *p* < 0.001. Kaiser and Rice (1974) proposed that, when KMO > 0.8, the significance of the value of Bartlett’s Test of Sphericity *p* < 0.05 was suitable for factor analysis. Accordingly, the maximum variance rotation method of analysis was used to obtain the rotation component matrix, which revealed four factors with eigenvalues greater than 1. Subsequently, two items with factor loadings less than 0.3 were deleted (“I am good at turning challenges into opportunities” and “I like to challenge the status quo”). The factor loading of the four factors was between 0.410 and 0.852, which fulfilled the standard criterion of the factor loading, i.e., greater than 0.3 (Zaltman and Burger, 1975). The explained variance ratio of proactive personality was 13.03%, social capital 13.41%, human capital 12.89%, and entrepreneurial intention 18.28%. The cumulative explained variance ratio of the questionnaire was 55.65%.

**Table 1:** Summary of the Exploratory Factor Analysis.

Item	Proactive personality	Social capital	Human capital	Entrepreneurial intention
Proactive Personality (explained variance ratio = 13.03%)				
1. I am always looking for a better way	0.595			
2. I tend to face a challenge directly.	0.510			
3. If I believe in an idea, nothing can stop me from realizing it.	0.410			
4. If I firmly believe in something, I tend to achieve it whatever.	0.452			
5. Nothing makes me more exciting than seeing my idea turned into reality.	0.551			
6. I am always looking for a new method to make my life better.	0.657			
7. I enjoy facing and overcoming difficulties.	0.587			
8. I am always hoping to be the special one in a group (perhaps in the whole world).	0.558			
Social Capital (explained variance ratio = 13.41%)				
9. I am skilled at solving problems with my classmates.		0.803		
10. I am able to share information with my classmates and learn from them.		0.752		
11. I am able to interact and exchange ideas with my families, teachers, and friends.		0.748		
12. I am able to develop solutions with my families, teachers, and friends.		0.682		
Human Capital (explained variance ratio = 12.89%)				
13. I believe I am highly skilled.			0.722	
14. I am widely considered the best in my profession.			0.712	
15. I am creative and bright.			0.576	
16. I am a professional in practice.			0.782	
17. I believe I can develop new ideas and knowledge.			0.551	
Entrepreneurial Intention (explained variance ratio = 18.28%)				
18. I am willing to work hard to become an entrepreneur.				0.731
19. My career goal is to become an entrepreneur.				0.813
20. I will work hard to start my own business.				0.797
21. In the future, I am ready to start my own business.				0.852
22. I sincerely consider starting my own business.				0.699
23. I strongly wish to start my own business.				0.817
The cumulative explained variance ratio = 55.65%				

### Reliability Analysis

This research used pilot test data to conduct reliability analysis. The result is as below: the proactive personality scale contained eight items and the Cronbach’s *α* = 0.796. The social capital scale contained four items, and the Cronbach’s *α* = 0.793. The human capital scale contained five items, and the Cronbach’s *α* = 0.811. The entrepreneurship intention scale contained six items, and the Cronbach’s *α* = 0.907, indicating that the reliability of each scale is quite good. In conclusion, this questionnaire includes four scales with a total of 23 items. The questionnaire’s total Cronbach’s *α* = 0.888, indicating that the reliability of the questionnaire is quite good.

## Results

### Measurement Model

This research used formal test data to CFA to test whether the measurement model, composed of four interrelated dimensions, including proactive personality, social capital, human capital, and entrepreneurial intention, fits. The data results of measurement model are as below: *χ*^2^ = 318.657, df = 224, *χ*^2^/df = 1.423, CFI = 0.977, GFI = 0.918, NFI = 0.926, RFI = 0.916, TLI = 0.974, IFI = 0.977, and RMSEA = 0.038, indicating that the measurement model and observation data fit well (McDonald and Ho, 2002). The Average Variance Extracted (AVE) and Construct Reliability (CR) of each dimension of the scales are as below: proactive personality AVE = 0.310, CR = 0.777; social capital AVE = 0.680, CR = 0.895; human capital AVE = 0.668, CR = 0.909; and entrepreneurial intention AVE = 0.589, CR = 0.896, where in the minimum of AVE is above 0.36, and CR is more than the standard of 0.6 (Fornell and Larcker, 1981), indicating that all dimensions have acceptable convergent validity.

### Common Method Variation

Multi-factor CFA and single-factor CFA were used for comparing the fit between the two and performing the CMV verification between the variables (Podsakoff and Organ, 1986). The results, which are listed in Table 2, revealed that the multi-factor model fitted well, with its χ^2^ being much lower than the single-factor model (*p* < 0.001), which implied that the two models were significantly different. Therefore, it was inferred that the common method bias was not serious in the present study.

**Table 2:** Comparison between the single-factor model and the multi-factor model.

Model	*χ* ^2^	df	Δ*χ*^2^	Δdf	*p*
Single-factor model	507.641	230	188.984	6	0.000
Multi-factor model	318.657	224			

### Descriptive Statistics and Correlation Analysis

This research used formal test data to perform Pearson correlation analysis to observe the correlation between variables. Table 3 shows that proactive personality and social capital have a significant and positive correlation (*r* = 0.614, *p* < 0.001); proactive personality and human capital have a significant and positive correlation (*r* = 0.740, *p* < 0.001); proactive personality and entrepreneurial intention have a significant and positive correlation (*r* = 0.724, *p* < 0.001); social capital and human capital have a significant and positive correlation (*r* = 0.638, *p* < 0.001); social capital and entrepreneurial intention have a significant and positive correlation (*r* = 0.665, *p* < 0.001); and human capital and entrepreneurial intention have a significant and positive correlation (*r* = 0.738, *p* < 0.001). The above correlation coefficients may be highly and moderately related, so it is necessary to test discrimination validity. The results are shown in Table 3. The number of the square root of AVE of each dimension greater than the correlation coefficient of each variables accounts for more than 75%, which meets the criteria for evaluation discriminant validity (Fornell and Larcker, 1981).

**Table 3:** Summary of the descriptive statistics and correlation analysis.

Variable	*M*	*SD*	Proactive personality	Social capital	Human capital	Entrepreneurial intention
Proactive personality	5.276	0.756	**0.557**			
Social capital	4.937	1.143	0.614^***^	**0.825**		
Human capital	4.581	1.075	0.740^***^	0.638^***^	**0.817**	
Entrepreneurial intention	4.181	1.047	0.724^***^	0.665^***^	0.738^***^	**0.767**

*** *p < 0.001*.

*Bolded fonts are AVE root values*.

### Structural Model

In this research, *t*-test and ANOVA analysis result show that gender (*t* = 1.785, *p* > 0.050) and major (*F* = 2.166, p > 0.050) have no significant difference in college students’ entrepreneurial intentions. Also, all college students that participated in this questionnaire survey took the innovation and entrepreneurship courses or entrepreneurship training activities in the college. Therefore, this research does not control the impact on entrepreneurial intention of gender, major, and whether participated in entrepreneurship courses or entrepreneurship training activities.

This research hypothesized proactive personality as the predictive variable, entrepreneurial intention as the dependent variable, and social capital and human capital as the mediating variable of the relation between proactive personality and entrepreneurial intention, and adopted Structural Equation Modeling (SEM) to explore the mediating effect of social capital and human capital. Based on the test procedures for mediating effect developed by previous researchers (Judd and Kenny, 1981; Baron and Kenny, 1986), firstly, the influence of proactive personality on entrepreneurial intention was explored. Second, both social capital and human capital were added as the mediating variables between proactive personality and entrepreneurial intention. If the path coefficient of the impact of proactive personality on entrepreneurial intention decreases, it demonstrates social capital and human capital play a partial mediating role. If the path coefficient of the impact of proactive personality on entrepreneurial intention is not significant, it demonstrates social capital and human capital play a complete mediating role.

**Figure 2:**
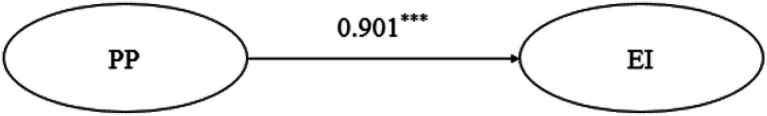
Main effect of the PP on EI. ^***^p < 0.001; PP, Proactive Personality; EI, Entrepreneurial Intention.

First, this research adopted SEM to construct the main effect model of the impact of proactive personality on entrepreneurial intention (as in Figure 2), and the model fitness index is: *χ*^2^ = 104.547, df = 76, *χ*^2^/*df* = 1.376, CFI = 0.983, GFI = 0.952, NFI = 0.939, RFI = 0.927, TLI = 0.979, IFI = 0.983, and RMSEA = 0.035. According to the research result, proactive personality can significantly predict college students’ entrepreneurial intentions (*β* = 0.901, *p* < 0.001), and proactive personality can explain 81.1% of the entrepreneurial intention, so H1 is supported.

**Figure 3:**
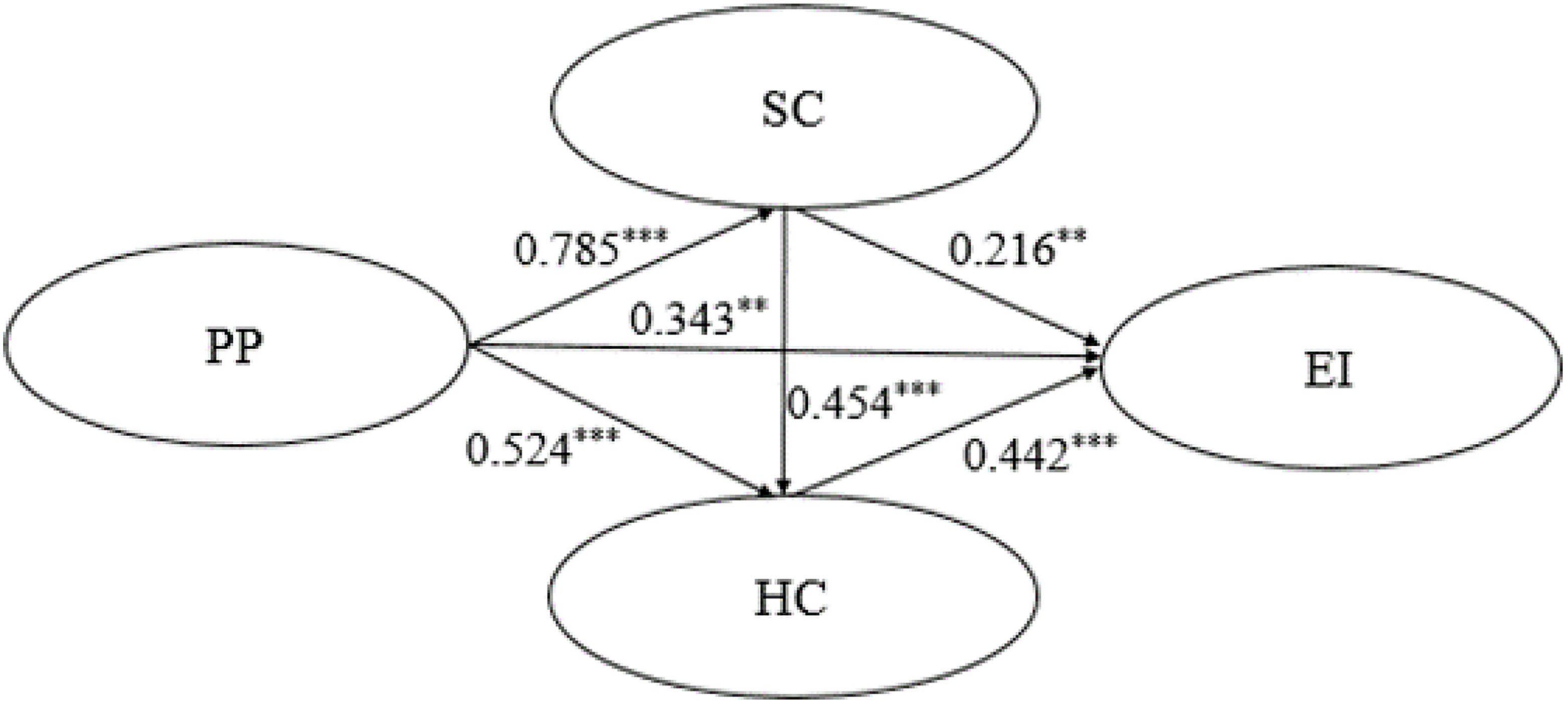
The chain mediation model. ^**^p < 0.01; ^***^p < 0.001; PP, Proactive Personality; EI, Entrepreneurial Intention; SC, Social Capital; and HC, Human Capital.

Second, this research adopted SEM to construct the chain mediation model (as in Figure 3). The model analysis results were as follows: *χ*^2^ = 318.657, *df* = 224, *χ*^2^/*df* = 1.423, CFI = 0.977, GFI = 0.918, NFI = 0.926, RFI = 0.916, TLI = 0.974, IFI = 0.977, and RMSEA = 0.038, indicating the chain mediating model fits well (McDonald and Ho, 2002).

In the path of proactive personality→social capital→human capital→entrepreneurial intention (see Table 4; Figure 3), proactive personality has a significant and positive impact on entrepreneurial intention (*β* = 0.343, *p* < 0.010); proactive personality has a significant and positive impact on social capital (*β* = 0.785, *p* < 0.001); social capital has a significant and positive impact on entrepreneurial intention (*β* = 0.216, *p* < 0.010); proactive personality has a significant and positive impact on human capital (*β* = 0.524, *p* < 0.001); human capital has a significant and positive impact on entrepreneurial intention (*β* = 0.442, *p* < 0.001); social capital has a significant and positive impact on human capital (*β* = 0.454, *p* < 0.001), It can be seen that after adding social capital and human capital as the mediating variables between proactive personality and entrepreneurial intention, the path coefficient of the impact of proactive personality on entrepreneurial intention decreased from 0.901 to 0.343, which is still significant. This indicates that social capital and human capital play a partial mediating role between proactive personality and entrepreneurial intention, which also preliminary verifies that social capital and human capital have a chain mediation effect in the relationship between proactive personality and entrepreneurial intention.

**Table 4:** SEM analysis results.

Path	*β*	SE	C. R.
Proactive personality→entrepreneurial intention	0.343^**^	0.201	3.207
Proactive personality → social capital	0.785^***^	0.200	7.850
Social capital→entrepreneurial intention	0.216^**^	0.074	2.757
Proactive personality →human capital	0.524^***^	0.199	5.490
Human capital→ entrepreneurial intention	0.442^***^	0.110	3.626
Social capital → human capital	0.454^***^	0.080	5.906

** *p* *< 0.01;*

*** *p < 0.001*.

*β*, *standardized coefficients; SE, standard error; and C. R., critical ratio*.

According to the suggestion of Nevitt and Hancock (2001), the method of Bootstrapping can be further used to test the stability of the mediating model. As an intensive computer-based statistical technique, the method of Bootstrapping reconstructs new samples representing the distribution of maternal samples by repeated sampling on limited sample data (Zhu, 1997). In order to test the chain mediation effect of proactive personality on entrepreneurial intention in college students, the present study employed Bootstrapping method which randomly repeated sampling 5,000 times. The results of this study should improve the accuracy of statistical estimates. Table 5 shows the analysis result of the chain mediating model using Bootstrapping method. Figure 3 shows the chain mediating model’s path diagram and effect size. It can be seen from Table 5 that the total indirect effect was 0.559. The total indirect effect was made up of three effects: indirect effect path 1: proactive personality→social capital→entrepreneurial intention (Indirect effect 1 = 0.170, LLCI = 0.340, and ULCI = 0.744), indirect effect path 2: proactive personality→human capital→entrepreneurial intention (Indirect effect 2 = 0.232, LLCI = 0.085, and ULCI = 0.410), indirect effect path 3: proactive personality→social capital→human capital→entrepreneurial intention (Indirect effect 3 = 0.157, LLCI = 0.033, ULCI = 0.291). The CIs of the above indirect effect exclude 0, showed that the three indirect effects all significant; moreover, proactive personality had a significant and positive prediction effect on entrepreneurial intention (Direct effect = 0.343, LLCI = 0.150, ULCI = 0.615). It showed that social capital had a partial mediation effect on the influence of proactive personality on entrepreneurial intention, human capital had a partial mediation effect in the influence of proactive personality on entrepreneurial intention, social capital and human capital had a chain mediating role in the influence of proactive personality on entrepreneurial intention. Therefore, H2–H4 of this research was once again confirmed.

**Table 5:** Mediation effects with bootstrapping.

Path	Effect	SE	95% LLCI	95% ULCI
Direct effect	0.343	0.040	0.150	0.615
Total indirect effect	0.559	0.101	0.340	0.744
Indirect effect 1	0.170	0.077	0.022	0.326
Indirect effect 2	0.232	0.085	0.085	0.410
Indirect effect 3	0.157	0.067	0.033	0.291

*Bootstrapping random sampling 5,000 times; SE, standard error; LLCI, Lower limit of confidence interval; ULCI, Upper limit of confidence interval; Indirect effect 1, proactive personality*→*social capital*→*entrepreneurial intention; Indirect effect 2, proactive personality*→*human capital*→*entrepreneurial intention; and Indirect effect 3, proactive personality*→*social capital*→*human capital*→*entrepreneurial intention.*

## Discussion

According to H1, the result of this research shows that college students’ proactive personality has a significant and positive impact on their entrepreneurial intentions, which is similar to the previous research result, indicating that the higher level of proactive personality is, the higher degree of entrepreneurial intentions will be (Zareieshamsabadi et al., 2010; Paul and Shrivatava, 2016). This is also similar to the research result in the educational field, which is that as college students’ proactive personality improves, their entrepreneurial intentions will increase, too (Basar, 2017; Zeb et al., 2019; Hossain and Asheq, 2020). Tan et al. (2021) noted six types of entrepreneurs’ personality traits: the need for achievement, risk-taking propensity, innovativeness, proactiveness, empathy, and moral obligation, and only four of them influence social entrepreneurial intention: proactiveness, innovativeness, empathy, and moral obligation. Differing from the study of Tan et al. (2021), the present study aimed to explore the influence of proactive personality on college students’ entrepreneurial intention and contributed to entrepreneurship research. Therefore, this research reveals this positive relationship under higher education background, which corresponds to the former research result. It is because college students with a higher level of proactive personality are more willing to take the initiative to change and chose to start their own business when they encounter unsatisfactory employment or working conditions. In contrast, college students with a lower level of proactive personality will only choose to adapt to the environment passively instead of entrepreneurship (Campbell, 2000). It can be seen that college students with a higher level of proactive personality, more spontaneity, and better ability to deal with changes and focus on the future will have higher entrepreneurial intentions.

According to H2, social capital has a partial mediation effect on the relationship between college students’ proactive personality and their entrepreneurial intentions. This result is the same as that of previous research, which is that the higher level of the proactive personality will cause more social capital (Thompson, 2005; Yang et al., 2011), and an increase in social capital will enhance the formation of entrepreneurial intention (Lee et al., 2014; Mahfud et al., 2020). Therefore, social capital plays an important mediating role between proactive personality and entrepreneurial intention. It is because that entrepreneurship requires support from the social network (Bian and Ang, 1997), and college students with a higher level of proactive personality are more likely to ask for others’ help to enlarge their social capital (Zhang et al., 2021) and thus enhance their entrepreneurial intentions. College students with a higher level of proactive personality can improve their entrepreneurial intentions by increasing social capital (Subramaniam and Youndt, 2005).

According to H3, this research confirms that human capital has a partial mediating role between college students’ proactive personality and entrepreneurial intentions, consistent with previous studies’ findings. It was demonstrated that the higher the level of proactive personality, the richer is the human capital (Seibert et al., 1999; Demirtas et al., 2017). The increased inhuman capital would, in turn, promote the development of entrepreneurial intention (Moradi et al., 2014; Miao et al., 2015). Therefore, human capital plays an important mediating role between proactive personality and entrepreneurial intention. It is because that this research believes that entrepreneurship requires a high level of knowledge and skills (Markman and Baron, 2003), and college students with a higher level of proactive personality are more likely to take the initiative to accumulate skills, experience, and knowledge (Subramaniam and Youndt, 2005). This will help solve complex problems during the entrepreneurial process (Gao et al., 2020) and increase their entrepreneurial intentions.

According to H4, this research further discovered the chain mediating role of social capital and human capital in the relationship between college students’ proactive personality and entrepreneurial intentions. This research result is similar to that of previous research, which is that social capital and human capital both have an important mediation effect on personality traits and entrepreneurship (Hsiao et al., 2016). Moreover, social capital improves entrepreneurial intention through human capital (Wang, 2021). This research result also verifies the view that social capital has a significant influence on the human capital formation (Coleman, 1988). Social capital is a drawing force, contributing to the creation and accumulation of human capital (Leana and Pil, 2006). Social capital is good for acquiring, transferring, or sharing human capital (Hansen, 1999). A high level of social capital will cause more frequent interaction among social members, which is beneficial to the generation of new human capital (Suseno et al., 2020). In this view, Chinese college students with more social capital will have more human capital. Also, the entrepreneurial intentions of Chinese college students with a higher level of proactive personality will be promoted by the chain mediation effect of social capital and human capital. Based on China’s cultural background, “Guan xi” is important, unique, and common in Chinese society (Zhang, 2006). “Chinese-style relationship,” such as relatives or acquaintances, plays an important role in personal career development and job search process (Bian, 1997; Farh et al., 1998; Chen and Chen, 2004; Zhang, 2006). Therefore, this research believes that Chinese college students’ social capital will significantly influence their human capital. The higher level of Chinese college students’ proactive personality will better increase their social capital and promote the accumulation of their human capital, enhancing their entrepreneurial intentions. In other words, college students with a higher level of proactive personality will have more social capital and human capital, facilitating the generation of their entrepreneurial intentions.

## Theoretical Contributions

This research result provides certain theoretical contributions to higher education literature. First, college students’ proactive personality has a significant and positive influence on entrepreneurial intentions. Second, social capital and human capital both have a partial mediation effect on college students’ proactive personality and entrepreneurial intentions. Third, social capital and human capital play a chain mediating role in the relationship between proactive personality and entrepreneurial intention. Although previous research has confirmed that proactive personality has a significant and positive impact on entrepreneurial intention (Zareieshamsabadi et al., 2010; Paul and Shrivatava, 2016), and the empirical research result exploring college students’ proactive personality and entrepreneurial intentions have been widely supported (Zeb et al., 2019; Hossain and Asheq, 2020), there were still a few discussion on the influence of proactive personality on the mediating mechanism of entrepreneurial intention. Although some previous empirical research took social capital theory and human capital theory as theoretical background at the same time, discussing the mediating role of social capital and human capital between employees’ internal control personality and entrepreneurship, and had obtained verification (Hsiao et al., 2016). The empirical research taking Chinese college students as research objects and exploring the mediating mechanism of proactive personality on entrepreneurial intention is still to be further discussed. Therefore, the contributions of this research lie in that it discovers that Chinese college students with a higher level of proactive personality will show more entrepreneurial intentions. Further, social capital and human capital can play an effective mediating variable effect between proactive personality and entrepreneurial intention. This research result enriches the relationship between college students’ proactive personality and entrepreneurial intentions under higher education background, which also promotes the application value of social capital theory and human capital theory in higher education.

## Practical Contributions

This research result also provides some useful, practical suggestions. First, since college students’ proactive personality has a significant and positive impact on their entrepreneurial intentions, there are several ways in terms of specific practices: colleges can conduct an assessment of proactive personality and promote college students’ active consciousness; colleges can carry out psychological quality training classes by introducing situational teaching to strengthen students’ willing, and increase college students’ proactive personality level.

Second, since college students’ social capital has a partial mediation effect between proactive personality and entrepreneurial intentions, increasing college students’ social capital cannot be ignored. Therefore, colleges can take measures from the following aspects: organize various club activities to provide support for college students on their interpersonal relationship; colleges can establish entrepreneurial information platforms so that college students’ entrepreneurial relationships can gradually form, which will be convenient for college students to find suitable partners or entrepreneurial mentor rapidly; it is also strongly recommended that colleges establish cooperation relationship with enterprises of different industries in various fields *via* integration of college and enterprises and in the mode of integration of industry and education, to promote college students’ social capital.

Third, this research result also shows that college students’ human capital partially mediates between proactive personality and entrepreneurial intentions. Therefore, it is suggested to take the following measures: at the same time opening entrepreneurship courses, colleges should also focus on developing entrepreneurship guidance and practice to increase students’ entrepreneurial knowledge and skills. Colleges can invite entrepreneurs and college students to deliver lectures to share entrepreneurial experiences with college students. Colleges can hold activities such as entrepreneurship simulation and entrepreneurship competition to train college students’ ability to solve problems related to entrepreneurship by allowing them to participate the simulation of real entrepreneurial issues to promote college students’ level of human capital.

Finally, this research reveals that college students’ social capital and human capital play a significant chain mediating role in the relationship between proactive personality and entrepreneurial intentions. Therefore, if colleges wish to ensure college students’ high level of proactive personality to acquire more entrepreneurial intentions, it is necessary to increase college students’ social capital and human capital. Social capital can positively influence human capital, and a higher level of human capital can help college students generate more entrepreneurial ideas to promote their entrepreneurial intentions. The chain mediating model of this research has a certain degree of practical contributions.

## Limitations and Future Direction

There are still several limitations in this research. First, only students in a college in Hebei, China were involved as volunteers of this research, limiting the result’s universality. Future research should expand the sample scope and explore college students from various cultural backgrounds from different countries. Second, this research only discusses social and human capital as mediating variables between college students’ proactive personality and entrepreneurial intentions. It still needs further exploration in the future whether there are more mediating variables in the process of influence or whether other variables moderate mediating variables. Furthermore, this research cannot make causal inferences of the nature of cross-section data, so it is suggested that future research adopt longitudinal study, time series cross-lag analysis, or rigorous quasi-experimental design to understand further the dynamic process of changing relationships between variables.

## Data Availability Statement

The raw data supporting the conclusions of this article will be made available by the authors, without undue reservation.

## Ethics Statement

The studies involving human participants were reviewed and approved by Hengshui University. The patients/participants provided their written informed consent to participate in this study. Written informed consent was obtained from the individual(s) for the publication of any potentially identifiable images or data included in this article.

## Author Contributions

Y-FL conceived the study idea, edited the data, performed the analysis and interpretation, drafted the skeleton of the manuscript, and critically reviewed the manuscript. JH contributed to constructing the model, interpretation of model results, and intensively editing the language of the manuscript. SG participated in the revision of the manuscript. All authors contributed to the article and approved the submitted version.

## Funding

This study was supported by Hebei Province Development of Human Resources and Social Security, China (Research on entrepreneurial intention management of College students in Hebei Province from the perspective of human capital; Project ID. JRS-2022-1037).

## Conflict of Interest

The authors declare that the research was conducted in the absence of any commercial or financial relationships that could be construed as a potential conflict of interest.

## Publisher’s Note

All claims expressed in this article are solely those of the authors and do not necessarily represent those of their affiliated organizations, or those of the publisher, the editors and the reviewers. Any product that may be evaluated in this article, or claim that may be made by its manufacturer, is not guaranteed or endorsed by the publisher.
